# “It’s a priority”: a qualitative analysis of the implementation of a maternal equity safety bundle in Massachusetts

**DOI:** 10.1186/s43058-025-00703-2

**Published:** 2025-03-27

**Authors:** Anna K. Daoud, Elysia Larson, Tonia J. Rhone, Claire R. Conklin, Heather Olden, Kali Vitek, Howard Cabral, Eugene DeClercq, Ndidiamaka Amutah-Onukagha, Hafsatou Diop, Audra R. Meadows

**Affiliations:** 1 The Perinatal Neonatal Quality Improvement Network of Massachusetts, Boston, MA USA; 2 Tufts University School of Medicine, Boston, MA USA; 3 NewYork-Presbyterian/Weill Cornell Medical Center, 525 E 68th St, New York, NY 10065 USA; 4 Beth Israel Deaconess Medical Center, Boston, MA USA; 5 Harvard Medical School, Boston, MA USA; 6 University of California—San Diego Medical Center, La Jolla, CA USA; 7 Harvard T.H. Chan School of Public Health, Cambridge, MA USA; 8 Boston University School of Public Health, Boston, MA USA; 9 Massachusetts Department of Public Health, Boston, MA USA

**Keywords:** Equity, Maternal morbidity, Quality improvement, Maternal safety bundles, Qualitative methods, Perinatal quality collaboratives, Consolidated framework for implementation research (CFIR)

## Abstract

**Background:**

Black-White inequities in severe maternal morbidity in the United States are extreme and growing. Maternal safety bundles (MSBs) have been associated with closing racial equity gaps in maternal health in some states. The objective of this study was to explore clinician perspective and experiences of implementing an Equity maternal safety bundle across five hospitals in Massachusetts to address inequities in perinatal care and birth outcomes.

**Methods:**

Focus group discussions and interviews were conducted in Fall 2022 and Fall 2023 (before and after Equity MSB implementation) among obstetric nurses, resident physicians, and attending physicians. Discussions were facilitated using a semi-structured guide developed using the Consolidated Framework for Implementation Research (CFIR). Transcripts were independently coded by two analysts using NVivo 14. A codebook was developed using CFIR for deductive coding. We added inductive codes as appropriate. We calculated Cohen’s kappa coefficients to assess interrater reliability. Themes were generated through an iterative process and compared across study time points.

**Results:**

Fifteen clinicians participated at each time point with similar distributions across race, ethnicity, gender, and profession. Seven themes emerged from these interviews: 1) the importance of leadership support to prioritize equity, 2) a culture of equity as a facilitator for implementation, 3) the need for improved processes for self-reported race, ethnicity, and language data collection, stratification, and dissemination, 4) staff, time, and funding as necessary resources, 5) the need for an early focus on staff education, 6) existing siloes between physicians and nurses and exclusion of trainees as barriers to implementation, and 7) differences between an Equity-MSB and other MSBs.

**Conclusions:**

Leadership prioritization of equity and a culture of equity emerged as facilitators to successful implementation of elements of the Equity MSB. Challenges identified included resistance to change among colleagues, limited resources, and clinician siloes. When compared to previously implemented MSBs, participants found that leadership made this work a priority. As future hospital teams embark on implementing equity-focused action, these known facilitators and barriers should be considered and addressed during the pre- and early-implementation phases.

**Supplementary Information:**

The online version contains supplementary material available at 10.1186/s43058-025-00703-2.

Contributions to the literature

- Quality improvement in obstetrics is a growing area of interest and promise for achieving maternal equity.
- This study adds to the paucity of literature on systems approaches to reducing maternal inequities using implementation science in obstetrics.
- Obstetric clinicians defined a culture of equity as individualized, evidence-based care that accommodates each patient’s needs and preferences while acknowledging existing inequities and providing a sense of belonging.
- Unlike other patient safety bundles, participants highlighted unique barriers to the Equity safety bundle including clinician defensiveness and difficulty measuring culture shift. We can address some of these barriers through education on anti-racism, implicit bias, and racial inequities in the pre-implementation phase.

## Background

Rates of maternal morbidity and mortality in the United States (US) are grossly inequitable, with non-Hispanic Black (NHB) birthing people having a maternal mortality rate 2.6 times greater than the rate of non-Hispanic White birthing people in 2021 [1]. Nationally, NHB individuals are also significantly more likely to experience severe maternal mortality (SMM) than NHW individuals (risk ratio 1.2 [95% CI 1.2–1.3] per 10,000 delivery hospitalizations; *P*< 0.001) [2]. Rates of severe maternal morbidity (SMM) in Massachusetts have risen from 1998 through 2018 for all racial and ethnic groups, with Black individuals experiencing the greatest absolute increase from 69.4 per 10,000 deliveries in 1998–2000 to 173.7 per 10,000 deliveries in 2016–2018, compared to a rise of 36.8 per 10,000 deliveries to 92.3 per 10,000 for all racial and ethnic groups in the same time periods [3]. Studies that control for education, income, and insurance type have demonstrated that despite controlling for these sociodemographic factors, disparities in rates of maternal mortality persist [4–8]. Interpersonal and structural racism are two contributing factors to these inequities that must be addressed [9, 10].

Maternal safety bundles (MSBs) are a collection of best practices aimed to address the leading causes of maternal morbidity and intended to be implemented using quality improvement (QI) methodology [11, 12]. Groups in California, Pennsylvania, and Texas have shown that maternal safety bundles can reduce racial inequities through quality improvement projects targeting diagnosis-specific morbidity such as postpartum hemorrhage [13–15]. Based on expert qualitative interviews and literature review, the Perinatal-Neonatal Quality Improvement Network of Massachusetts (PNQIN) [16], the Massachusetts state perinatal quality collaborative (PQC), developed a Maternal Equity Safety Bundle (Equity MSB) and implementation toolkit based on content from the Alliance for Innovation on Maternal Health (AIM)’s Reduction of Peripartum Racial and Ethnic Disparities Conceptual Framework and Maternal Safety Consensus Bundle [17, 18]. PNQIN’s Equity MSB aimed to assist Massachusetts hospital teams in eliminating racial inequities in SMM in the state by identifying a list of structures to have in place, including standards for staff training in antiracism, best data practices, and evidence based processes for care delivery, that can improve equity in maternal health and tools that aid in implementation of these structures.

This study aimed to explore clinician perspective and experiences of implementing a maternal safety bundle on equity across five hospitals in Massachusetts to address inequities in perinatal care and birth outcomes. The focus on health equity is particularly significant, addressing a timely and critical issue by examining efforts to eliminate racial inequities in maternal health outcomes with real world relevance to guide organizations facing similar challenges.

## Methods

This is a prospective qualitative study analyzing the implementation of a quality improvement project that took place across five hospitals in Massachusetts. The study follows the Standards for Reporting Qualitative Research (SPQR) checklist [19], completed in Appendix E.

### Bundle implementation

Five hospitals were selected from the 40 birthing hospitals in Massachusetts using criterion purposive sampling for participation in safety bundle implementation and interviews for the “Be A Mom” Study (R01 MD016026) at Tufts University. Bundle implementation was conducted in partnership with PNQIN. Criteria for hospital selection included: participation in PNQIN perinatal quality initiatives, providing care to NHB individuals in Massachusetts, and training residents in Obstetrics and Gynecology. Together, the five hospitals selected provide birth care to greater than 50% of NHB birthing people in MA. Characteristics of included hospitals can be found in Appendix A. From September 2022 through October 2023, PNQIN facilitated the implementation process with an Equity MSB Toolkit, monthly webinars on bundle topics, data review, and quarterly coaching sessions with participating hospitals. The Equity MSB includes a set of 10 measures and a toolkit to accompany and aid in implementation of these measures [17]. This MSB was developed through a rigorous literature review followed by qualitative interviews with experts in maternal health equity and a modified Delphi process. This is the first study to evaluate the experience of clinician’s implementing of the Equity MSB, though components of it include evidence-based best practices including timely treatment of severe hypertension in pregnancy and use of quantitative blood loss measurement [20, 21]. Other components of the MSB are recommended best practices to advance health equity such as data disaggregation and clinician anti-racism training [22, 23]. The monthly webinars were held virtually open to all clinicians at participating hospitals. Examples of review topics included methodology for collecting self-reported race and ethnicity data, guidelines for timely treatment of hypertension, and how to create team equity goals and antiracism statements. Coaching sessions were held virtually for one-hour quarterly with one participating team at a time to discuss progress in Equity MSB implementation and included institutional SMM review stratified by race and ethnicity with comparison to state SMM rates. Individualized, hospital SMM data were reviewed during these coaching calls, as well as any difficult topic areas of interest. Each site had one member of their team, either a nurse or attending physician, submit structure, process, and outcome measures monthly through RedCap [24]. Bundle measures can be found in Table 1.

**Table 1:** PNQIN equity maternal safety bundle measures

Structure Measures
1. Does your hospital have a formal equity team based in obstetrics/reproductive health that includes diversity of roles, race/ethnicity, and community member?
2. Has your hospital developed and communicated their obstetric equity goals to the perinatal faculty and staff including SMART or AIM equity goals, an antiracism statement, and change statements?
3. Does your hospital collect race, ethnicity, and language data upon registration/entry for obstetric care? Is it collected through self-report?
4. Does your hospital stratify process and outcome data by race, ethnicity, and language?
5. Has your hospital adapted and implemented a Patient Reported Experience Measure (PREM)?
Process Measures
1. Among your OB staff (physicians, midwives, nurses, etc.) how many completed a training program on implicit bias, racism, and racial disparities in the past 2 years (training must include education on implicit bias and racial disparities, e.g. SPEAK UP)?
2. Among the number of birthing people with persistent (twice within 15 min) new-onset Severe HTN [(Systolic: ≥ 160 or Diastolic: ≥ 110), excludes birthing people with an exacerbation of chronic HTN], how many were treated within 1 h with IV Labetalol, IV hydralazine, or PO Nifedipine?^a^
3. Among the birth admissions, how many had a hemorrhage risk assessment completed with a risk level assigned?^a^
4. Among the birth admissions, how many had measurement of blood loss from birth through the recovery period using quantitative and cumulative techniques?^a^
Outcome Measure
1. Severe maternal morbidity (SMM) rates by race and ethnicity

^a^Stratified by race/ethnicity for non-Hispanic White and non-Hispanic Black

### Participant selection

At each participating hospital the department chair identified a labor and delivery (L&D) nurse(s), OB/GYN attending physician(s), and OB/GYN resident physician(s) to form an implementation team. Each hospital received $12,000 annually to implement the MSB across 36 months. These funds could be used at the discretion of the study team. Hospitals participated in implementing three MSBs in succession: Obstetric Hemorrhage (June 2021 – November 2021) [25], Severe Hypertension in Pregnancy (January 2022—June 2022) [26], and PNQIN Maternal Equity (Equity MSB, September 2022- October 2023) [17]. Each team member was invited to complete a structured survey and participate in a focus group discussion (FGD) before and after implementing each MSB. For the PNQIN Equity MSB, team members participated in either a FGD or interview post-implementation.

### Focus groups

FGDs or qualitative interviews were conducted in Fall 2022, before Equity MSB implementation, and Fall 2023, following Equity MSB implementation. All site team members, physicians and nurses, were eligible to be included in focus group discussions (FGDs) and qualitative interviews. Most team members completed the year-long implementation, however, several positions changed due to turnover and residency graduation. At the pre-implementation timepoint, we conducted three focus groups. Focus groups were conducted with clinicians from all five hospitals, separated by role (nurses, resident physicians, attending physicians). This grouping aimed to capture role-specific insights while maintaining diversity in institutional contexts. In the post-implementation timepoint, a FGD was conducted amongst the participating nurses from the same five participating hospitals, and individual interviews with the remainder of the participants due to scheduling conflicts. Each FGD had four to six participants and lasted 75–90 min. The individual interviews had one participant and lasted 45–60 min. All participants completed a self-reported demographics survey via RedCap at the start of the FGD or qualitative interview [27, 28]. All participants provided verbal informed consent at the beginning of the FGD. FGDs were conducted online and audio-recorded using Zoom and professionally transcribed using Rev software [29, 30]. Transcripts were reviewed for accuracy by the research team, comparing the text to the original audio files. Participants were given a $40 gift card for their time.

### Implementation science framework

This study followed the Consolidated Framework for Implementation Research (CFIR). CFIR is a framework to understand the context and identify areas that may strengthen and hinder successful implementation, previously applied to evaluate the implementation of other safety bundles [31, 32]. We applied the CFIR framework in data collection, analysis, and interpretation to understand determinants of implementation, and themes in the experience of clinicians implementing the PNQIN Equity MSB across five hospitals in Massachusetts [33]. The FGD and qualitative interview facilitator used a semi-structured guide for the pre-implementation interviews (Appendix B) and post-implementation interviews *(*Appendix C) that was developed by adapting questions from the updated CFIR and Interview Guide Tool associated with the original 2009 CFIR [32, 34, 35]. Most of the questions came from the “Inner Setting” and “Intervention Characteristics” domains, followed by the “Outer Setting”, “Implementation Process”, and “Characteristics of Individuals”, respectively. Questions from within each domain were chosen for their anticipated likelihood of identifying modifiable barriers and facilitators as well as relevance to the stage of Equity MSB implementation for each organization. An initial codebook was created using the CFIR Codebook Template based on the 2009 framework and updated using the updated CFIR and its intersectionality supplement [32, 36, 37].

### Qualitative analysis

Focus group transcripts were uploaded, coded, and categorized using NVivo 14 qualitative software [38]. Transcripts were independently coded by two authors (AD and TR) deductively using the initial CFIR-based codebook. Over iterative reviews of the transcripts, inductive codes were added appropriately and mapped onto the CFIR framework. The codebook was refined iteratively throughout the coding and consensus processes. The final codebook can be found in Appendix D. Cohen’s kappa coefficients were calculated to assess inter-rater reliability for each transcript included. The Cohen’s kappa coefficient was 0.83 (range 0.72–0.88) indicating excellent agreement [39].

Codes were organized by CFIR domain before placing them into larger categories that were then converted into themes. Themes were generated by examining both associated codes and quotes for each CFIR construct. Themes across the two-time points (pre- and post-implementation) were compared.

This study was approved by the Tufts University School of Medicine institutional review board as #MOD-23-STUDY00001400.

## Results

Three FGDs were conducted in the pre-implementation time point, and one FGD and nine qualitative interviews were conducted in the post-implementation time point. A total of fifteen clinicians across the three clinician groups participated at each time point with similar distributions across race, ethnicity, gender, and profession (Table 2). The majority of participants have spent greater than five years at their respective institutions. We present the findings of pre- and post-implementation FGDs and interviews according to the salient CFIR domains and sub-domains, before drawing attention to the seven themes that we generated across the data set. Select participant quotes by CFIR domain can be found in Table 3.

**Table 2:** Focus group discussion and qualitative interview participant demographics

			Pre-Implementation (*N* = 15)	Post-Implementation (*N* = 15)
Gender	Female		13 (86.7%)	14 (93.3%)
	Male		2 (13.3%)	1 (6.7%)
Race and Ethnicity	White		13 (86.7%)	10 (66.7%)
	Black or African American		0 (0.0%)	2 (13.3%)
	Asian		1 (6.7%)	1 (6.7%)
	Hispanic		1 (6.7%)	2 (13.3%)
Specialty	Nurse		6 (40.0%)	6 (40.0%)
	Physicians		9 (60.0%)	9 (60.0%)
		Attending Physicians	5 (55.6%)	4 (44.4%)
		Resident or Fellow Physicians	4 (44.4%)	5 (55.6%)
Years at the Hospital Site	Less than 5 years		6 (40.0%)	6 (40.0%)
	5–10 years		3 (20.0%)	4 (26.7%)
	More than 15 years		6 (40.0%)	5 (33.3%)

**Table 3:** Select participant quotes by CFIR domain

CFIR Domain	Adapted CFIR Code	Definition	Quote
Characteristics of Individuals	Self-Efficacy	Individual belief in their own capabilities to execute courses of action to achieve implementation goals	“I feel very confident. Our senior leadership is very much prioritizing this work and is available to provide whatever resources our group wants.”—Nurse, Pre-Equity
			“I don’t know that we’ll ever be completely implemented. I think that this is going to be one of those things that we were just continually working on. It’s going to be a 10-to-15-year journey before we can look back and say, look at what a good job we did.”—Attending Physician, Pre-Equity
			“Very confident because that’s one of the things that attracts me as well to this position.”—Nurse, Post-Equity
	Facilitators & Barriers	Factors that support or give the participants a lack of confidence in successfully implementing the MSB	“I think one of the issues that I noticed is that things are pretty siloed. For example, we had Grand Rounds recently highlighting some of the equity bundles, the plans and current stats, and that was very much physician heavy. I think it’s a little challenging for me to really understand what the onboarding process is for nursing.” – Resident Physician, Pre-Equity
			“I think we already had buy-in as part of our institutional identity, that we believe in trying to be leaders in that space, but also having institutional buy-in ahead of time and just believing it was the right thing to do, having multiple people within leadership who believed it was the right thing to do.”—Attending Physician, Post-Equity
	Individual Motivators	The motivations of the individual participants to take part in the Equity MSB implementation	“I don’t think it’s fair that Black women are dying more.”—Attending Physician, Post-Equity
			“I mean, selfishly as a woman of color, it’s like, "This is what we need to do." And even my experiences as a provider being a person of color, it’s just something that you deal with every day. And so I do think something that requires attention and intention.”—Resident Physician, Post-Equity
Inner Setting	Inner Culture	Norms, values, and basic assumptions of a given organization and how these aspects impact implementation	“I think that we all have our implicit biases and so it does play a role in the culture as well.”—Resident Physician, Pre-Equity
			“I have a very anti-change site that is being forced to change a lot, and so we’re getting better at change. I think the culture, either half our culture’s going to be super psyched with it and the other half is going to be a little bit more resistant.”—Attending Physician, Pre-Equity
			“Culture is just the hardest. Again, a lot of us aren’t trained well to do that. Like everyone else said it’s bigger than just implementing an order set on L&D or having a hemorrhage readiness kit for the OR. It’s so much bigger and broader that I think it’s just difficult. I don’t know what needs to be changed but it’s just so big it feels less concrete and harder to do.”- Attending Physician, Pre-Equity
			“The culture is huge. I mean all of the population that we serve here, and that people generally work here because they’re very motivated by wanting to promote equity and serving the underserved patient.”—Nurse, Pre-Equity
			“A culture of equity is where we design and build all of our care to accommodate the preferences and needs of every patient as they come through our care. We work to understand where things need to be different for different people or groups, and equally important for all people that we do things well in whatever way that means to reach the goal, which is a healthy birthing person and a healthy infant and a healthy family.”—Attending Physician, Post-Equity
			“A culture of equity means that there is a shared mental model that all patients deserve equitable care and that doesn’t mean that each patient gets the same type of care. But considering where the patient is and then understanding that that might mean one patient needs a certain amount of care but to strive for the same outcomes. And for that to be a priority equally no matter who the patient is.”—Resident Physician, Post-Equity
			“I think there’s been a real culture shift around equity work, particularly in the last three years. And it helps support and propel this type of work.”—Attending Physician, Post-Equity
	Implementation Climate	The absorptive capacity for change, shared receptivity of involved individuals to an innovation, and the extent to which use of that innovation will be rewarded, supported, and expected within their organization	“It’s a priority.”—Nurse, Pre-Equity
			“Just like everybody, we’ve had massive turnover and I think half of our staff are under five years. And this is completely anecdotal, but I observe that most, many of our younger staff are more receptive to change. And there’s a lot of pushback from our expert level, more seasoned nurses. Not all of them, but some of them. We’ve always done it this way and this has been working fine, why do we have to change it? And a lot of disheartening amount of negativity about different changes that we’ve tried to implement. Especially from people who’ve been doing it a certain way for decades.”—Nurse, Post-Equity
			“It’s a smaller group, but it’s enough that though negativity is contagious. And so personally, I am mindful of who I’m choosing to train my new nurses and making sure that it’s not one of those people who’s resistant to change, to be honest, even though they may have a lot of expertise, because I don’t want some of the resistance or negativity to spread more than it needs to.”—Nurse, Post-Equity
	Structural Characteristics	The social and physical architecture and size of an organization, suggested changes to accommodate implementation, and the level of resources organizational dedicated for implementation and on-going operations including physical space and time	“So we hired an OB outcomes nurse about 11 months ago. We had been wanting to do a more comprehensive data dashboard for a long time, but just didn’t have the manpower. So it’s now up and running, and [redacted] has been able to stratify, I believe, all of the monthly data that he’s reporting out on by race and ethnicity, and we’re working on adding language to that.”—Nurse, Post-Equity
			“I feel like in order for a resident to walk away from their million and one tasks and be able to dedicate the time that the patient deserves through TeamBirth, someone needs to be answering those pages, somebody needs to be doing that.”—Resident Physician, Post-Equity
	Readiness for Implementation	Tangible and immediate indicators of organizational commitment to its decision to implement an innovation	“I think it only helped the conversation. I think it only demonstrated that there was broad support for it, and that many people expressed the desire to do this type of work, but there was a lack of either a team or infrastructure or support.”—Attending Physician, Post-Equity
			“I feel a little more surprised that people scored highly on the readiness, but again, I don’t know exactly what goes into the score. I think I feel more surprised that people scored highly on readiness for equity, but I don’t know that it should be differently measured or different.”—Resident Physician, Post-Equity
Outer Setting	Influences to Implementation	External factors that influenced the decision to implement the Equity MSB	“I think hospitals are paying attention to this right now and in some ways, we’re ahead of other service lines in thinking about the outcomes our patients have and how disparate they are. I think it’s given us a little bit of an opportunity to lead.”—Attending Physician, Pre-Equity
			“I think data is a big reason. The data is pretty compelling and PNQIN has worked nicely to share each center’s own SMM data by race, which people often think bad things happen in the world but at other places, not at their own facilities. Then you look at your own data and you’re like wow, that stuff’s happening here.”—Attending Physician, Pre-Equity
Intervention Characteristics	Suggested Changes, Adaptability & Design Quality	Perceived excellence in how the innovation is bundled, presented, and assembled, the degree to which an innovation can be adapted, tailored, refined, or reinvented to meet local needs, and suggested changes to improve the intervention	“I think each one of these bullets is its own big QI implementation to really do it.”—Attending Physician, Pre-Equity
			“I think all the tools and all the parts of the implementation are needed and necessary.”—Nurse, Post-Equity
			“I think that the parts that have been easier to do and that I also think are more crucial are a lot of the data stuff, so being able to stratify the data, being able to present the real data, having dashboards, measuring quality within that scope. So I think those things, in my mind, at least, are the core to the bundle, that if it wasn’t there, it wouldn’t seem complete.”—Attending Physician, Post-Equity
			“I just have one little piece to say about doulas. There’s some grant going on where we’re going to have some doulas that are going to be assigned to patients that are in the community women’s center. And so we are trying to work with them to sort of build a relationship with nursing because we’ve had some instances where there have been doulas from the outside that have come in, that there’s been a little bit of friction between nursing and the doulas. Feeling like the doulas are being obstructionist a little bit with the care of the patient. So I think that’s one area that we need to work on, that relationship between doulas and nursing, to provide better care for our patients.” – Nurse, Post-Equity
	Complexity	Perceived difficulty of the innovation, reflected by duration, scope, radicalness, disruptiveness, centrality, and intricacy and number of steps required to implement	“It felt less concrete than the hemorrhage and hypertension bundles. And so that’s like, for hemorrhage, do you have an emergency hemorrhage kit on the floor? Do you have a patient education packet to give to patients on discharge? I can create this document, I can send it to patients, I can create this package, have it on labor and delivery. I can create this hypertensive order set and have it in Epic. But a lot of community engagement. I don’t even know how to define it, how to do it. It’s not something that on our day-to-day things that we do practically as a QI team or as physicians do well or have the tools or know how to do it. So some of them were very concrete, like some of the data stuff, even some of the PREMs, but a lot of the other stuff seemed really soft, and so there was less guidance and less practical ways to implement things.”—Attending Physician, Post-Equity
			“I think part of the challenge as a resident trainee in this process in comparison to the other bundles is that it was a little harder to tell what the implementation itself was. I do know that there’s certain things that have occurred since the start of the implementation. But for example, when we were talking about the hypertensive and postpartum hemorrhage, it was very actionable, very checklist, very part of the order system. You had to order meds. You had to write down the QBL. It was something that was just very concrete versus this has just been challenging to know exactly what it means.”—Resident Physician, Post-Equity
	Evidence Strength & Quality	The kind of supporting evidence or proof is needed about the effectiveness of maternal safety bundles to get staff on board, including involvement of co-workers and administrative leaders	“Continued data generation and continued attention to it by leadership… You can do all the QI in the world, you can have the best tool and the best even implementation of something, but if the people on the leadership on the floor and the people on the floor don’t prioritize it or don’t find it as important as other things, it’s not going to happen. So I would say mid-level leadership is key.”—Attending Physician, Post-Equity
Implementation Process	Reflecting & Evaluating	Quantitative and qualitative feedback about the progress and quality of implementation accompanied with regular personal and team debriefing about progress and experience	“I just think that we’ve done three bundles in rapid succession, that I feel like we’ve sort of taken one piece of each of them that we’ve been working on. It’s felt a little piecemeal. To be able to do it well from start to finish with stakeholder engagement and driver diagrams and all of that…that’s a three-month process. I haven’t been able to do it for this bundle yet, nor did we do it for hypertension, frankly. We just did interventions.”—Attending Physician, Pre-Equity
			“I do think that sometimes there’s a little bit of a disconnect between the nursing staff and the MD staff and when things might work best for them from a practical workflow perspective and when things might work best for the resident who’s holding three pagers and in triage and everywhere else. I think that could have been a little bit smoother, especially talking to a lot of my juniors about their experience, they were like, "I kind of wish someone had talked to me first about what time might’ve been better to do it.”—Resident Physician, Post-Equity
			“I think it went well because we had very motivated onsite people to get the implementation going. I think just the mindset of the department we work in has been very in tune with this, and we really want to make it better.”—Attending Physician, Post-Equity
			“That’s a pinpoint dynamic, but I think that’s true with PQCs across the board. If you look at who participates, it’s primarily nursing and that is not a group that is holding the power at most hospitals, the purse strings.”—Attending Physician, Post-Equity
	Executing	Carrying out or accomplishing the implementation according to plan	“It was hard, but once we put that anti-racism statement out, everybody was jumping on board, but it was hard to get everybody to endorse it and to say, "Yes, this is where we had to do it." It was like a constant, come on, we got to get this going. Oh, somebody else has to okay it. Oh, branding has to okay it. Oh, communications has to okay it. So it’s always something. But then finally I was gone by the time it was posted, but everybody was, Nurse C had said, that everybody’s on board by then, and maybe it’s just a time for that to get that endorsement. It would’ve been easier for us to do the work if that was clear right from the start.”—Nurse, Post-Equity
			“We are still building our patient voice. We have one identified patient who is recently joining us in some meetings, but really looking to expand that as a known, really strong and necessary voice. We do have a doula that’s on that committee. So we are really trying to fan out the voices of the people that we care for in a way that’s thoughtful. Again, lots of work to do for sure, but our work thus far has been really collaborative.”—Nurse, Post-Equity

### Characteristics of individuals

#### Self-efficacy

Participants were asked at both timepoints to describe their level of confidence for successfully implementing the Equity MSB and to discuss the reasons for that level of confidence. Self-efficacy was evaluated by the two independent coders using categories of “positive self-efficacy,” “negative self-efficacy,” and “neutral self-efficacy” to describe each individual participant’s as with significant confidence in succeeding in implementation, a lack of confidence in succeeding in implementation, and neutral opinion regarding ability to succeed in MSB implementation, respectively. Pre-implementation, participants predominantly reported feeling confident in successfully implementing the Equity MSB, while few reported feeling a lack of confidence and neutral regarding successful implementation. Resident physicians described feeling neutral amounts of confidence or lack of confidence in successful bundle implementation at the pre-implementation time point, notably because they were not sure what success would be and what the components of the bundles were. One resident physician noted that success would be more qualitative or based on a “gut feeling” regarding a culture shift compared to other bundles defining success with specific clinical measures. However, at the post-implementation time point, there was greater consensus in feeling confident in completing and sustaining the implementation — only two attending physicians reported feeling neutral, whereas all other participants reported significant confidence. Participants explained their confidence or lack thereof based on available perceived facilitators and barriers at their respective institutions (Table 4).

**Table 4:**
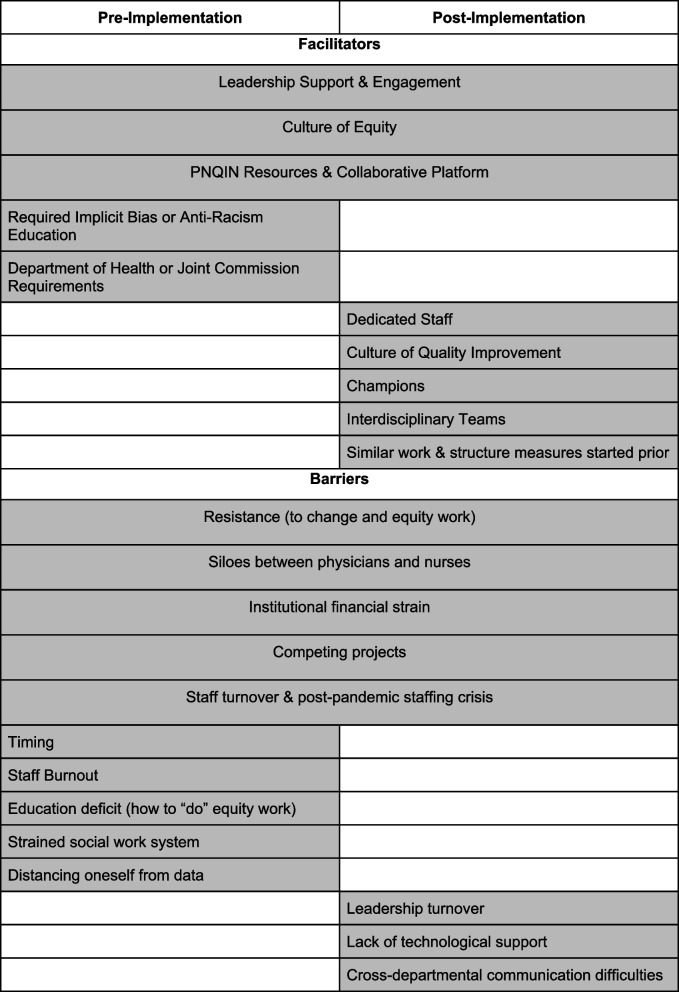
Identified facilitators and barriers for self-efficacy in implementation

#### Perceived facilitators & barriers

Perceived facilitators for successful implementation amongst participants across both time points were a culture of equity and leadership support. Perceived barriers to success across both time points were resistance to implementation of equity projects, siloes between nurses and physicians, and competing interests, such as other equity-focused projects.

#### Individual motivators

Participants described their personal motivations for involvement in the Equity MSB including reducing racial inequities, personal experiences as women of color, this project aligning with their moral values, media on the Black Maternal Health crisis, and inclusion of equity in mandatory orientation. Resident physicians discussed individual motivations more than the other two groups, though participants from all three clinician groups discussed the topic. Three resident physicians discussed personal experiences as women of color motivating them to go into medicine to eliminate inequities for patients and providers.

### Inner setting

#### Inner culture

Across time points, participants discussed needing culture change for this bundle, rather than solely practice change. Participants emphasized the importance of culture as both a facilitator and a barrier to implementation. The most common topic discussed around culture was a culture of equity. Participants from all three clinician groups discussed colleagues being motivated to improve equity and being mission-driven in caring for underserved populations as part of an existing culture of equity. This was noted as a strength for implementation, but participants cautioned that it was important to merge enthusiasm with action. In the pre-implementation time point, anti-racism or implicit bias education was emphasized as a necessary mechanism for advancing a culture of equity, rather than a culture of “blame and shame.”

Participants at the post-implementation time point were explicitly asked to define a culture of equity. Most commonly a culture of equity was described as individualized care that is designed and built to accommodate needs and preferences of each patient. They described improved access to care and care that addresses social determinants of health, acknowledges implicit biases, and acknowledges that disparities exist and the institution cares about eliminating them. Other common terms used to define a culture of equity included being welcoming and open to all people, providing respectful care, providing “gold standard” care, providing a sense of belonging, humility, anti-racism, and good communication. Participants noted that a culture of equity required a culture shift that has only recently occurred at participating institutions. An example of an effective culture shift at a participating institution is the use of identity pronouns. A resident physician noted they felt there was a culture of equity among the resident physicians but was unsure this was the case for the entire department.

Participants described leadership, resident physicians, and nurses as responsible for establishing culture, though nurses were most often acknowledged as culture drivers. Leadership pushed for culture change by prioritizing equity work. Department cultures resistant to change and embedded with implicit biases were implementation barriers. Select quotes on Inner Culture can be found in Table 3.

Participants also discussed a culture of implicit bias and differential treatment, which they noted was largely due to experiences with nursing and separation of private attending physician patients and resident physician patients. One resident physician noted a disconnect between the education they receive through their residency program and the culture practiced at their institution. One attending physician noted that they practice at a site resistant to change, including difficulty in developing and embracing a culture of equity. Participants shared anecdotes of clinicians becoming defensive and taking discussions around equity work personally.

#### Implementation climate

During the pre-implementation timepoint, the participants highlighted the importance of education on anti-racism, implicit bias, and obstetric inequities as part of the learning and implementation climate, with specific examples of training. The attending and resident physicians discussed receiving their training as part of grand rounds and resident education but felt that this education was siloed from the nurses and medical assistants. The nurses discussed SPEAK UP training and onboarding training in equity as a priority set by nursing leadership [40]. SPEAK UP was discussed by several nurses as a positive environment to learn about obstetric racism for this bundle.

There was agreement across both time points that equity-focused projects were a relative priority within their institutions due to leadership endorsement. There was consensus that either equity was already a priority set by departmental and institutional leadership or that it should be. Most nurses agreed senior leadership at their respective institutions make equity a priority. By making this project a priority, participants noted it helped overcome the barrier of competing projects.

Several participants across all three clinician groups discussed resistance to change both for projects impacting workflow and specifically around racial equity work. Implicit bias, emotional reactions to being accused of inequitable care, and examples of differential treatment were discussed across both time points, though more examples were given in the post-implementation time point. All three clinician groups described instances of pushback from nurses due to a lack of education on inequities, defensiveness surrounding inequitable care amongst physicians and nurses, and “older” staff being less accepting of equity work and more resistant to change. Two participants shared that despite barriers, staff are overall receptive, and they attribute this to the sociopolitical beliefs in this geographic region and a high level of turnover amongst nurses such that most nurses are younger and more receptive to change. One resident physician shared that faculty have accepted the bundle, but that it was not disseminated to private practices.

#### Structural characteristics

All three clinician groups emphasized the desire for an easier process of accessing data, stratifying data by race/ethnicity, and sharing data with the interdisciplinary team on L&D. In the pre-implementation time point, lack of ease of accessing data and lack of staff time were the most common structural barriers discussed. Post-implementation, participants reported an increase in available staff time for data management and developing interdisciplinary teams through study participation.

### Outer setting

#### External policies and incentives

The most common motivating factor for engaging in this MSB pre- and post-implementation was knowledge of state and national inequities in maternal health outcomes. Pre-implementation, leadership and individual-level financial incentives (paid staff time) were other affirmative influences, while post-implementation, hospital-level financial incentives and previous PNQIN participation were the second most common influences discussed. Examples of hospital-level financial incentives included the cost of morbidities, pay-for-performance models, and reduced malpractice costs. For nurses at the pre-implementation time point, the COVID-19 pandemic served as a barrier to the implementation of initiatives due to burnout.

### Intervention characteristics

#### Areas for improvement & design quality

The most common suggestions for improvement from the pre-implementation cohort were a longer timeframe for implementation, a multidisciplinary education team to train all staff on the bundle and improving access to institutional data. Suggestions for improvement in the post-implementation cohort included an improved readiness assessment with an environmental scan by PNQIN, simulations, publicizing institutional outcome data, and requiring documentation of completed structure and process measures in patient charts. While many participants in the post-implementation cohort said they would not change any components of the bundle, some participants across both time points found the Equity MSB as “vague” compared to the other MSBs and suggested a checklist or a different form of itemized guidance for the intervention to be more objective.

Participants additionally discussed interventions to complement the Equity MSB with the most common being TeamBirth [41–43]. Doulas, equity-focused morbidity and mortality conferences, an equity accelerator, and social determinants of health screening were suggested across both time points. However, in the post-implementation time point, a common suggestion was to improve interpreter services and to have patient-family advocates as part of the team, a measure of the Equity MSB.

#### Complexity

Across both time points, participants noted that the Equity MSB, unlike the obstetric hemorrhage and timely treatment of hypertension MSBs they previously implemented, was more difficult to measure because it requires “culture shift” and could evoke emotional reactions and defensiveness, particularly addressing racism. Participants shared that this bundle differs from the other two because it is not isolated to the L&D setting and is more interdisciplinary. A participant noted that, unlike quantitative blood loss (QBL), bias is not as objectively measurable. Trainees noted the MSB was vague compared to other bundles and less objective, stating that this bundle requires all new protocols and systems unlike the other bundles which worked well into their workflow.

### Implementation process

In reflecting and evaluating their implementation experience, participants discussed successes over the year of implementation in the post-implementation time point. Successes included collecting and stratifying data by race, ethnicity and language (ReaL) by patient self-report, creating equity dashboards, forming and sustaining an equity team despite staff and leadership turnover, and sustaining shared decision-making practices learned from participating in TeamBirth concurrently. Challenges were discussed and included an inadequate timeframe with rapid succession of implementing three bundles, difficulty understanding all the separate components of the Equity MSB, pushback due to the topic, siloes between physicians and nurses, and gaps in language equity tools. A participant noted that outcomes would be truly seen in 10 to 15 years, and they did not expect immediate improvements in outcomes with this bundle to drive motivation. Participants raised concerns in the post-implementation time point on sustainment once PNQIN oversight and funding ends. One attending physician discussed financial pressures as limiting implementation, and they describe the system now as focused on clinical productivity. When discussing implementation of the structure measures of the bundle, participants voiced the most difficulty with adding a patient representative to their equity team.

Participants also described drivers in the implementation process. Across both time points, the most common type of supporting evidence on the Equity MSB effectiveness was leadership support and endorsement. Participants included examples of leadership engagement in the day-to-day of the bundle implementation (attending webinars, dissemination of equity goals). Participants additionally discussed nursing champions as providing supporting evidence to frontline staff and individual requirements from the Joint Commission aligning with components of the bundle as strengths in the evidence for implementing this bundle.

Differences between physicians and nurses in the context of implementation of the Equity MSB were discussed by all three clinician groups. One attending physician highlighted the disparity between who does the work with this bundle, which includes a large proportion of women and nurses, and who sits in leadership positions in obstetric departments. However, another participant shared that the primary pushback they receive on equity work is from nursing due to what they hypothesize to be related to anti-racism and equity-related educational gaps. Nurses described physicians doing equity work that does not include them and is likely less effective due to this silo. Trainees described siloes in workflow between nurses and resident physicians and gave an example of how the residents’ schedule was not taken into consideration for meetings regarding TeamBirth.

## Discussion

**Fig. 1:**
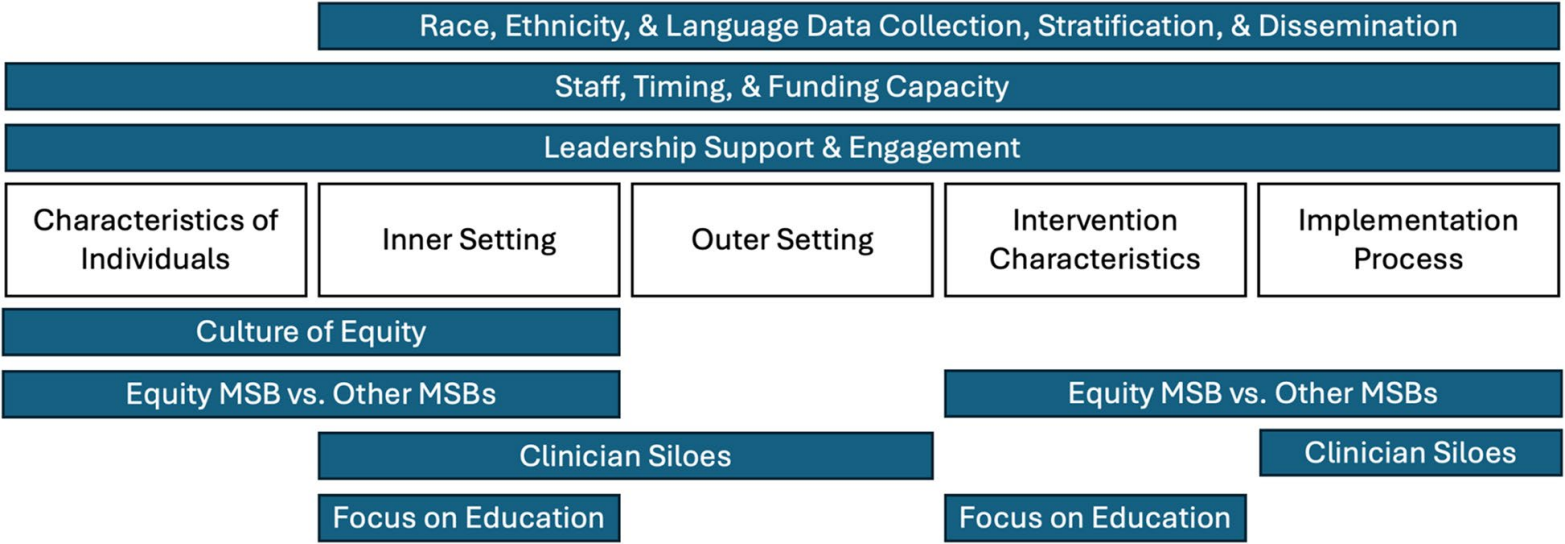
Application of themes across CFIR domains

This study examined the experiences of clinicians implementing an Equity MSB across five participating hospitals in Massachusetts with a goal of identifying themes and actionable suggestions for implementation of equity-focused QI projects. Seven themes emerged under the CFIR determinants across both timepoints: 1) the importance of leadership support and engagement, 2) a culture of equity as a facilitator for implementation, 3) the need for improved processes for self-reported race, ethnicity, and language data collection, stratification, and dissemination, 4) staff, time, and funding as necessary resources, 5) the need for an early focus on staff education, 6) existing siloes between physicians and nurses and exclusion of trainees as barriers to implementation, and 7) the difference between an Equity-MSB and other MSBs (Fig. 1). These seven themes serve as both facilitators and barriers, and are most important to optimize for sites to implement the Equity MSB effectively.

Previous MSB evaluations in the literature have examined the implementation of initiatives on obstetric hemorrhage, vaginal breech deliveries, placenta accreta spectrum, surgical site infections, preventing obstetric anal sphincter injuries, severe hypertension, and obstetric early warning systems. The CFIR framework was previously applied to identify how implementers responded to an obstetric hemorrhage initiative and identified determinants adversely impacting implementation, such as project complexity [31]. Qualitative methods have been applied to identify facilitators and barriers of safety bundles on vaginal breech deliveries and found that clinicians believed facilitators included individual capabilities and multidisciplinary team support, while barriers included lack of resources and social obstacles [44]. The literature on MSB evaluations additionally includes results of quantitative methods including bundle compliance, process measure utilization, staff participation, and health outcomes such as rates of transfusion [45–49]. Several equity-focused projects in maternal health have been implemented and evaluated, including the impact of establishing a system-wide team goal of reducing maternal morbidity for Black individuals [14]. In this initiative, multidisciplinary site based teams were established, conducted case reviews of SMM impacting Black individuals, and evidence-based practices were selected and implemented based on individual site case reviews, which resulted in significant reductions in maternal morbidity for Black individuals from pre-goal to post-goal development at most of the system sites [14]. A hospital implementing the obstetric hemorrhage bundle found that by stratifying their data by race and ethnicity, presenting it to hospital clinicians, and selecting QI strategies based on the disparities, they were able to significantly decrease SMM from hemorrhage for Black individuals [15]. This study builds on this existing literature by identifying factors that can be optimized and barriers that must be addressed for successful implementation of equity-focused QI projects in the obstetric setting to eliminate inequities in maternal health. By utilizing qualitative methods, the perspectives of frontline clinicians can be amplified to better understand the implementation process and experience.

### Leadership prioritization and support

Leadership emerged as a crucial factor for implementing the Equity MSB under the CFIR determinant of *Inner Culture*. Participants emphasized leadership support as essential to overcoming barriers to implementation. Departmental prioritization of equity facilitated successful implementation of governance, clinician education, and data infrastructure, which are key strategies to promote maternal equity through perinatal quality [50]. Our findings align with previously identified organizational factors and practices that distinguish high from low-performing hospitals including leadership support and engagement, strong physician-nurse communication and teamwork, sharing performance data with frontline data, and explicit awareness of racial inequities and the impact has on adverse outcomes through a culture of equity [51]. A session of leaders in Maternal Fetal Medicine implementing an obstetric early warning system across the country also found that successful implementation required administrated and leadership support, evaluation of hospital culture, and coordination of interdisciplinary teams [52]. Participants in this study described a strong sense of confidence (high levels of self-efficacy) for successfully implementing this intervention, which they attributed to leadership support and an institutional culture of equity. These factors made this project a priority at the participating institutions, helping to overcome some of the predicted and existing barriers to implementation.

### Organizational culture

The culture of healthcare institutions can influence implementation. In this qualitative study, participants highlighted institutional culture as both a facilitator and barrier. Culture is a modifiable organizational factor that affects quality, outcomes, and care delivery processes [53, 54]. Previous studies in obstetrics have shown that institutional culture affected outcomes such as rates of cesarean delivery [55, 56]. In this study, participants named cultures of equity, accountability, and quality improvement as facilitators when implementing equity-focused projects. Participants defined a culture of equity as one that provides individualized, evidence-based care that accommodates each patient’s needs and preferences while acknowledging existing inequities and fostering a sense of belonging. Leadership priorities and staff turnover over the past three years have contributed to a culture shift towards equity. Leadership can facilitate culture change by prioritizing specific, as was done with the Equity MSB [57]. Additionally, siloes between physicians and nurses were identified as a barrier to implementation and successful equity work, whereas interdisciplinary teams in the post-implementation were highlighted as facilitators of successful implementation. These findings suggest that future equity-focused QI projects should target culture change through leadership endorsement, incorporating education on this topic at faculty and staff orientation, encouraging interdisciplinary collaboration, and building a culture of accountability with a positive learning climate. This approach, as supported by previous literature on perinatal QI, is preferred over a culture of “blame and shame” and can lead to sustainable improvement in maternal equity [58–60].

### Perinatal quality collaborative (PQC) support

The Perinatal Neonatal Quality Improvement Network of MA (PNQIN), state’s Perinatal Quality Collaborative or PQC, was identified as a facilitator to and influence for implementation. PQCs have been successful in engaging teams to use perinatal QI to eliminate racial inequities, and this study demonstrated their positive role as a facilitator for hospital teams implementing MSBs [13, 15]. The PQC team’s process that developed the Equity MSB identified several foundational components for equity work: 1) governance, 2) prioritization from leadership, 3) antiracism and bias education and training, 4) data disaggregation by race, ethnicity, and language (REaL), and 5) patient voice and community engagement [17]. Among these, community engagement is essential for ensuring that equity initiatives are informed by and responsive to the needs of the most affected [50]. In this study, participants found community engagement, specifically, adding a community member or patient representative to their equity committee, as the most challenging structure measure to implement. Perceived barriers included lack of finances to support this person’s time and travel, a lack of approved processes, and uncertainty about how to effectively engage with the community. Building community engagement can be further addressed and supported by hospital-level patient and family advisory councils and by state PQCs through statewide patient and family advisory councils. There is need for further literature on community engagement in maternal health QI, with a current study ongoing to observe the impact of a hypertension MSB on community engagement [61].

### Motivations to engage

Individual participants discussed the effect of intersectionality on their experiences with the Equity MSB when describing individual motivators and the extent to which the MSB meets the needs of the patients they serve. Previous literature identified that personal experience as a minority was a significant motivator for physicians to engage work reducing health disparities [62, 63]. While participants discussed how this project would benefit the patients they serve, they noted it would not address each form of discrimination by itself, citing a need to complement the Equity MSB with tools addressing interpersonal racism such as patient advocates and improved tools for language equity for patients with limited English proficiency. Inequities in maternal health are driven by different forms of racism including structural, institutional, and interpersonal racism [9]. While this MSB targets inequities at the structural and institutional levels, it only begins to address interpersonal racism through implementing a process measure for the percentage of obstetric staff that have completed an anti-racism training in obstetrics. Overall, our ability to apply the outer systems and structures construct described in the intersectionality supplemented CFIR and tool was constrained by limited discussion during the FGDs [36].

### Complexities of equity-focused action

The complexity of the intervention, combined with the one-year timeframe, and pre-existing inner culture of the institutions, affected the ease and success of implementation. One study applied the CFIR framework to the evaluation of a state-wide obstetric hemorrhage initiative found that participants implementing that project also reflected positively on the project, but its complexity adverse impacted implementation and required additional efforts to improve the effectiveness of the implementation process [31]. Although the Equity MSB followed a similar framework and layout as previous MSBs adapted from AIM, participants found this bundle to be more difficult to understand, implement, and measure. Previous projects on evaluating equity-based QI projects demonstrated successful reduction of disparities following the implementation of a larger equity focus, with site specific initiatives based on their institution’s disparity data [14, 15]. The goal of the Equity MSB was to establish the structure for individual hospital’s to move forward with individual initiatives based on their inequities once able to stratify their data effectively by race and ethnicity, using the process measures on timely treatment of hypertension and obstetric hemorrhage as examples. However, the concurrent focus on both these structure and process measures, could have adversely impacted the complexity.

Factors that limited individuals’ confidence in implementation were largely resource-based, including access to data, time, finances, and staff, under the CFIR determinant of *Structural Characteristics*. Access to data, including the accurate collection of REaL data, the ability to stratify data by REaL, and to share this data through equity data dashboards was a common theme across both time points both as an area for improvement, but also as an area of success in the post-implementation time point. However, a unique barrier for this MSB was resistance to equity work and implicit biases of their colleagues. Anecdotes shared by clinicians across all three clinician groups indicated defensiveness in response to equity-focused QI projects, among small groups of clinical staff at their respective institutions. Despite evidence of inequities in medicine, some providers continue to deny racism in healthcare settings [64]. In this study, reports of defensiveness from participants are consistent with literature on previous perinatal equity QI projects, which suggest using an appreciative inquiry approach that focuses on collective rather than individual data, the historical roots of the inequities, and successes of the team [65, 66].

Resident physicians reported feeling less involved in this MSB implementation compared to other MSBs. Although the MSB was introduced with a toolkit that included steps for implementation, participants suggested creating a checklist for itemized guidance to make the intervention more objective. The measures and associated toolkit included specific structures to address equity, which were evaluated on a Likert Scale ranging from 1 (not in place) to 5 fully in place), with 2–4 describing various stages of progress. The clinical process measures were the same measures used in the two-prior implemented MSBs, with the addition of stratifying these measures by REaL. For all bundles implemented during this project, the outcome measure was SMM. Further research is necessary to explore the perception that the Equity MSB was complex, and less objective compared to other MSBs. Conversations about racism can evoke strong emotions and resistance, which could impact the perception and implementation of the intervention. The emotional and political nature of discussing equity, implicit bias, and racism, along with doubts about whether this intervention could lead to achieve maternal equity, may have contributed.

### Practical steps for implementation

To effectively apply an equity lens to QI projects, several practical steps can be taken based on our findings. First, securing leadership commitment and consistently communicating that equity is a priority are essential. Equity is foundational to healthcare quality [67]. Leadership support is crucial for building governance and ensuring the allocation of necessary resources, such as staff, time, and funding. Second, addressing resistance to change by promoting an appreciative inquiry approach and creating a supportive environment for learning and improvement is essential [65]. Educating and training staff on equity, antiracism, and implicit bias through comprehensive and continuous programs helps maintain awareness and develop the skills needed to address inequities. Encouraging interdisciplinary collaboration and communication across professional roles and involving trainees in equity projects is also important. Third, implementing robust systems for self-reporting of REaL demographics, confirming accurate data collection, and ensuring the analysis of REaL data across all maternity projects is a key step. This data should be used to identify disparities, inform practice changes, and track progress towards equity goals. Finally, fostering a culture of equity within the institution involves integrating equity into the department’s mission, values, and operations while conducting regular training and discussions on equity and inclusive excellence.

### Strengths and limitations

Our study had a number of strengths. First, we had interdisciplinary and diverse perspectives with resident physicians, attending physicians, and nurses through a longitudinal, implementation science process across one year. Our study emphasized interdisciplinary collaboration, communication, and practical recommendations for improving implementation of equity-focused projects. Another notable strength of our study is the structured approach guided by CFIR. This framework ensures a thorough and systematic analysis, enhancing the reliability and validity of our findings. Lastly, we oversampled hospitals that care for Black birthing people in Massachusetts to target our intervention to the most affected group.

Though this study followed rigorous qualitative methodology, there were limitations. First, the study team evaluating the implementation of the Equity MSB and conducting this analysis also designed and implemented this intervention. Because of this, study team members explicitly discussed their potential biases while developing codes and creating themes. They also looked for disconfirming data and included those findings in results. Second, there was limited selection of respondents using criterion purposive sampling, which can contribute to bias in their pre-existing knowledge on inequities in maternal care and personal motivations to address them. By interviewing only the attending physician, resident physician, and nursing leads at each site, we did not obtain the perspective of other stakeholders in the implementation process including patients, leadership, the anesthesia team, and reception. Community or rural hospitals were not included in this sample, limiting transferability, and the study analyzes the subjective experiences of obstetric clinicians in five urban academic medical centers in Massachusetts, which may not be applicable to other institutional settings. The five hospitals included in this study participated in at least two prior Maternal MSB implementation projects before Equity MSB implementation, such that staff at these institutions likely have increased awareness and buy-in to the Equity MSB compared to institutions new to MSBs. Most of the structure and process measures in the Equity MSB focused on the Labor and Delivery room setting, though inequities occur throughout the pregnancy and postpartum period for birthing period. Lastly, the findings from the pre-implementation FGDs were not used to adjust the implementation process during the yearlong implementation as the implementation process was set a priori by study design for the first year including in this analysis. This likely impacted the success of the implementation of this MSB over the first year studied. Findings from this analysis will be used to refine the implementation of this MSB and future MSBs by PNQIN.

## Conclusion

Participating hospitals successfully implemented equity-promoting structures through leadership by departmental equity teams and processes for stratifying quality data by demographics. This evaluation demonstrates the importance of establishing data and leadership infrastructure for applying an equity lens to all projects within obstetric units of academic hospitals serving NHB patients. Challenges identified included resistance to change, limited resources, and clinician siloes. Culture is a facilitator of change, and itself can be a target of interventions in hospitals and was identified as an important determinant to the implementation process. Leadership made this bundle a priority because of their focus on improving inequities in maternal care.

Our findings can inform the implementation of equity projects in maternal health. Lessons learned provide a roadmap for other institutions aiming to address maternal equity as well as to PNQIN for refinement of the Equity MSB to support community engagement and clinician defensiveness in equity efforts. PQCs can offer useful tools for this purpose. Our team encourages ongoing adaptation and research to continue improving applications of Equity MSBs.

## Supplementary Information

Supplementary Material 1.

Supplementary Material 2.

## Data Availability

The datasets used and/or analysed during the current study are available from the corresponding author on reasonable request. Associated materials are included in attached Appendices.

## Abbreviations

AIM: Alliance for Innovation on Maternal Health
CFIR: Consolidated Framework for Implementation Research
FGD: Focus Group Discussion
L&D: Labor and Delivery
MSB: Maternal Safety Bundle
NHB: Non-Hispanic Black
PNQIN: The Perinatal Neonatal Quality Improvement Network of Massachusetts
PQC: Perinatal Quality Collaborative
PREM: Patient Reported Experience Measure
QBL: Quantitative Blood Loss
QI: Quality Improvement
ReaL: Race, Ethnicity, and Language
SMARTIE: Specific, measurable, actionable, realistic, timely, inclusive, equitable
SMM: Severe Maternal Morbidity
US: United States
