# Supplementary material for: “It’s a priority”: a qualitative analysis of the implementation of a maternal equity safety bundle in Massachusetts
Source: Implement Sci Commun. 2025 Mar 27;6:28. doi: 10.1186/s43058-025-00703-2 (PMC11951730; doi:10.1186/s43058-025-00703-2)
Supplement: Supplementary file 1 — Supplementary Material 1. [file 43058_2025_703_MOESM1_ESM.docx]

**Appendix A. Characteristics of Included Hospitals**

|  | Delivery Volume (number deliveries/per year) | Urbanization Level | Teaching Hospital Status | Organization Status |
| --- | --- | --- | --- | --- |
| Hospital A | 2000-5000 | Urban | Teaching Hospital | Nonprofit |
| Hospital B | 2000-5000 | Urban | Teaching Hospital | Nonprofit |
| Hospital C | 5000-8000 | Urban | Teaching Hospital | Nonprofit |
| Hospital D | 2000-5000 | Urban | Teaching Hospital | Nonprofit |
| Hospital E | 2000-5000 | Urban | Teaching Hospital | Nonprofit |

**Appendix B. Pre-Implementation FGD Guide**

I. INTRODUCTION

**Purpose:**

Hello everyone. Thanks for joining our focus group today for the Be A Mom Study. My name is [NAME] and I’ll be leading this discussion today. We are here today to talk about your hospital implementation of the Equity maternal safety bundles as defined by the AIM Program.

Overall, we are working to ensure safe and equitable birth for all. There are no right or wrong or desirable or undesirable comments or answers. You can agree or disagree with each other, and you can change your mind, however, please respect one another’s opinions. We would like you to feel comfortable saying what you really think and how you really feel.

**Consent:**

Our group discussion with you and your colleagues will take approximately 90 minutes to complete.

Participation is completely voluntary. We will use the information from this discussion to guide future research and interventions. We will present this data without any details that could identify you. You are completely free to skip any question if you choose. If you choose not to participate, it will not result in any penalties or loss of benefits to you. If you withdraw or are withdrawn from the focus group any data collected from you before your withdrawal will still be used for the study.

We will audio record the discussion and have it transcribed. We will then destroy the audio recording. If any names are mentioned, they will be removed from the transcript. If you have questions or concerns, you can contact the co-investigator, Audra Meadows, MD, MPH at ameadows@pnqinma.org or the Tufts Health Sciences IRB (617)-636-7512. If you decide to participate, you will receive a $40 Target Gift Card after participating.

**Participant Introductions**

Now let’s start with a brief survey for our data collection – I will put the link in the chat here, please take one minute to fill out it out.

II. Intervention Characteristics

Thank you. Now I would like to start our discussion about the Equity maternal safety bundle as defined by PNQIN. I am going to share my screen briefly to show you the bundle I am talking about so that we are all on the same page: [see PNQIN one pager] As you can see, this bundle has novel structure measures with process measures that are found in the Obstetric Hemorrhage and Severe Hypertension in Pregnancy bundles. These process measures will be reported by race/ethnicity.

1. What kind of supporting evidence or proof is needed about the effectiveness of maternal safety bundles to get staff on board? Co-workers? Administrative leaders?

2. What kinds of changes or alterations do you think you will need to make to the maternal equity bundle so it will work effectively at your hospital sites? Probe further: Do you think you will be able to make these changes? Why or why not?

3. Are there components of the equity bundle that should not be altered? Which ones should not be altered? Probe: Why?

III. Outer Setting

4. How well do you think the implementation of the equity bundle will meet the needs of the patients at your site? In what ways will the intervention meet their needs? Potential probe: what additional intervention would complement the maternal safety bundles to meet their needs?

5. What influenced the decision to implement the intervention? Probe: local, state, or national performance measures, policies, regulations, guidelines, financial or other incentives?

IV. Inner Setting

6. What kinds of infrastructure changes at your hospital site will be needed to accommodate implementation of maternal safety bundles? [Probe whether there are changes needed that are specific to the hypertension bundle]

· Changes in scope of practice? Changes in formal policies? Changes in information systems or electronic records systems? Changes in resources? Other?

· What kind of approvals will be needed? Who will need to be involved?

· Can you describe the process that will be needed to make these changes?

7. How do you think the culture (general beliefs, values, assumptions that people embrace) at your site and also within the OBGYN department will affect the implementation of the maternal equity safety bundles?

· Can you describe an example that highlights this?

· To what extent are new ideas and culture shifts embraced and used to make improvements in your organization and the delivery of care for birthing persons?

8. What kinds of incentives are there to help ensure that the implementation of the maternal equity bundle is successful? What is your motivation for wanting to help ensure the implementation is successful? Can you think of other incentives that would ensure successful implementation of the equity bundle that we have not already discussed? Are the incentives different for different maternal safety bundles?

9. What level of endorsement or support have you seen or heard from leaders at your site to implement this equity focused maternal safety bundle? What kind of support or actions can you expect from leaders in your organization to help make implementation successful?

V. Characteristics of Individuals

10. How confident are you that your hospital team will be able to successfully implement the maternal equity bundle? What gives you that level of confidence (or lack of confidence)?

VI. Process

11. Will feedback be elicited from staff? From the individuals served by your organization? How and what kind of feedback?

VII. Conclusion

12. Does anyone have anything else they want to add to our discussion today?

This concludes our discussion for today. As a reminder, we would like to ask that each of you respect the privacy of everyone in this group by not using names when discussing the content of this discussion with others. Thank you very much for participating; I appreciate your time and your comments have been very helpful. Your input will directly lead to bundle toolkit and implementation improvement. Thank you again!

**Appendix C. Post-Implementation FGD Guide**

I. INTRODUCTION

**Purpose:**

Hello everyone. Thanks for joining our final focus group for the Be A Mom Study. My name is [NAME] and I’ll be leading this discussion today. We are here today to talk about your hospital implementation of the PNQIN Maternal Equity safety bundle.

Overall, we are working to ensure safe and equitable birth for all. There are no right or wrong or desirable or undesirable comments or answers. You can agree or disagree with each other, and you can change your mind, however, please respect one another’s opinions. We would like you to feel comfortable saying what you really think and how you really feel.

**Consent:**

Our group discussion with you and your colleagues will take approximately 90 minutes to complete.

Participation is completely voluntary. We will use the information from this discussion to guide future research and interventions. We will present this data without any details that could identify you. You are completely free to skip any question if you choose. If you choose not to participate, it will not result in any penalties or loss of benefits to you. If you withdraw or are withdrawn from the focus group any data collected from you before your withdrawal will still be used for the study.

We will audio record the discussion and have it transcribed. We will then destroy the audio recording. If any names are mentioned, they will be removed from the transcript. If you have questions or concerns, you can contact the co-investigator, Audra Meadows, MD, MPH at ameadows@pnqinma.org or the Tufts Health Sciences IRB (617)-636-7512. If you decide to participate, you will receive a $40 Target Gift Card after participating.

**Participant Introductions**

Now let’s start with a brief survey for our data collection – I will put the link in the chat here, please take three to five minutes to fill out it out.

II. Intervention Characteristics[AM1] [LE(O2]

Thank you. Now I would like to start our discussion about the PNQIN Maternal Equity safety bundle. I am going to share my screen briefly to show you the bundle I am talking about so that we are all on the same page: [see PNQIN Equity Bundle one pager of structure and process measures]. As you can see, this bundle has novel structure measures with process measures that are found in the Obstetric Hemorrhage and Severe Hypertension in Pregnancy bundles. These process measures will be reported by race/ethnicity.

1. What kinds of changes or alterations do you think are needed to make to the maternal equity bundle work effectively at your hospital sites? Probe further: Were you able to make these changes? Why or why not?

2. Are there components of the equity bundle that should not be altered? Which ones should not be altered? Probe: Why?

III. Outer Setting

3. How well do you think the implementation of the equity bundle will meet (or has met) the needs of the patients at your site? In what ways has the intervention met their needs? Potential probe: what additional intervention would complement this intervention to meet their needs?

4. What influenced the decision to implement the intervention (PNQIN maternal equity bundle)? Probe: local, state, or national performance measures, policies, regulations, guidelines, financial or other incentives?

IV. Inner Setting

5. Similar to our first question, what kinds of infrastructure changes at your hospital site were needed to accommodate implementation of the equity bundle? [Probe whether these needed changes were specific to the equity bundle]

· Changes in scope of practice? Changes in formal policies? Changes in information systems or electronic records systems? Changes in resources? Other?

· What kind of approvals were needed? Whose involvement was needed?

· Can you describe the process of the changes?

6. How do you think the culture (general beliefs, values, assumptions that people embrace) at your site and within the OBGYN department affected the implementation of the maternal equity safety bundle?

· Can you describe an example that highlights this?

· To what extent are new ideas and culture shifts embraced and used to make improvements in your organization and the delivery of care for birthing persons?

· What is a culture of equity to you?

7. What kinds of incentives or resources helped to ensure the bundle implementation was successful, or what incentives or resources were missing that you think would have helped ensure successful implementation?

a. What is your motivation for ensuring implementation success of this bundle? Are the incentives different for other maternal safety bundles?

8. What level of endorsement or support have you seen or heard from leaders at your site to implement this equity focused maternal safety bundle?

a. What kind of support or actions were received from leaders in your organization to help make implementation successful?

b. Probe if they say the level of support or endorsement was low: Are there examples that you can give that demonstrate lack of support

9. When reviewing team readiness scores using the ORIC survey we asked you prior to each bundle, we found that each team had a high level of readiness to implement change, and this did not change with more experience implementing a bundle. Do you think measuring or discussing “team readiness to implement change” supported equity bundle implementation? Was this the same or different from “team readiness” supporting implementation of the HTN or OB HEM bundles?

V. Characteristics of Individuals

10. How confident are you that your hospital team will be able to successfully complete implementation of the equity bundle? Sustain what has been implemented? What gives you that level of confidence (or lack of confidence)?

VI. Process

11. Was feedback elicited from staff? From the individuals served by your organization? How and what kind of feedback did you receive?

VII. Conclusion

12. Does anyone have anything else they want to add to our discussion today?

This concludes our discussion for today. As a reminder, we would like to ask that each of you respect the privacy of everyone in this group by not using names when discussing the content of this discussion with others. Thank you very much for participating; I appreciate your time and your comments have been very helpful. Your input will directly lead to bundle toolkit and implementation improvement. Thank you again!

**Appendix D. Qualitative Analysis Codebook**

| **Code Name (Adapted CFIR Code)** | **Description/Definition** |
| --- | --- |
| **Characteristics of Individuals** |  |
| Self-Efficacy | Individual belief in their own capabilities to execute courses of action to achieve implementation goals. |
| Incentives to ensure success | Extrinsic incentives such as goal-sharing, awards, performance reviews, promotions, and raises in salary, and less tangible incentives such as increased stature or respect. |
| Negative - lack of confidence | Participants describe a lack of confidence in implementing or completing implementation of the MSB. |
| Neutral regarding implementation success | Participants report feeling neutral regarding success of MSB implementation. |
| Positive - report confidence in implementing MSB | Participants describe feeling highly confident in implementing or completing implementation of the MSB. |
| Compatibility | The degree of tangible fit between meaning and values attached to the innovation by involved individuals, how those align with individuals’ own norms, values, and perceived risks and needs, and how the innovation fits with existing workflows and systems. |
| Influences to implementation | Factors that individuals believe will influence MSB implementation. |
| Barriers to bundle implementation success | Factors that give the participants a lack of confidence in successfully implementing the MSB. |
| Facilitators to bundle implementation | Factors that support confidence in successfully implementing the MSB |
| Individual motivations | The motivations of the individual participants to take part in the Equity MSB implementation. |
| Individual Stage of Change | Characterization of the phase an individual is in, as s/he progresses toward skilled, enthusiastic, and sustained use of the innovation. |
| Other Personal Attributes | A broad construct to include other personal traits such as tolerance of ambiguity, intellectual ability, motivation, values, competence, capacity, and learning style. |
| **Inner Setting** |  |
| Impact of culture | Norms, values, and basic assumptions of a given organization and how these aspects impact implementation. |
| Culture of Equity | Response to the question of “What is a culture of equity to you?” Describing the participants’ definitions of a culture of equity. |
| Leadership changes | The impact of changing leadership on the culture of an organization. |
| Implementation Climate | The absorptive capacity for change, shared receptivity of involved individuals to an innovation, and the extent to which use of that innovation will be rewarded, supported, and expected within their organization. |
| Learning Climate | Discussion on a climate that prioritizes education, in which there is time for reflective thinking and evaluation and psychological safety to try new methods. |
| Relative Priority | Individuals’ shared perception of the importance of the implementation within the organization. |
| Resistance to MSB implementation | Participants discussing environment of resistance to MSB implementation or contributing factors to the resistance. |
| Infrastructure changes | The social and physical architecture and size of an organization, suggested changes to accommodate implementation, and the level of resources organizational dedicated for implementation and on-going operations including physical space and time. |
| Approvals or department collaborations needed | Suggested approvals or interdepartmental collaborations are needed to implement changes to improve bundle success/implementation. |
| Electronic medical records | Changes in information systems and EMRs necessary or suggested to improve success/implementation of MSB. |
| Protocols and Policies | Changes in protocols (formal & other), changes in scope of practice to improve bundle implementation. |
| Staff capacity | Changes in staff capacity (number and time) to improve bundle implementation. |
| Readiness for implementation | Tangible and immediate indicators of organizational commitment to its decision to implement an innovation. |
| **Intervention Characteristics** |  |
| Benefits of MSBs | Stakeholders’ perception of the advantage of implementing the innovation versus an alternative solution. |
| Suggested Changes, Adaptability & Design Quality | Perceived excellence in how the innovation is bundled, presented, and assembled, the degree to which an innovation can be adapted, tailored, refined, or reinvented to meet local needs, and suggested changes to improve the intervention. |
| Trialability | The ability to test the innovation on a small scale in the organization, and to be able to reverse course (undo implementation) if warranted. |
| Autonomy or ownership of the process | Providers/staff feeling ownership of the implementation process as a change to facilitate implementation. |
| Components that should not be altered | Specific components of the equity bundle that should not be altered. |
| Specific suggested alterations | Suggestions made by participants on specific factors to change in the MSB. |
| Complexity | Perceived difficulty of the innovation, reflected by duration, scope, radicalness, disruptiveness, centrality, and intricacy and number of steps required to implement. |
| Innovation Source | Perception of key stakeholders about whether the innovation is externally or internally developed. |
| Interaction with other MSBs | Discussion on how the Equity MSB can interact with or complement the other MSBs implemented. |
| Interventions to complement MSB | Suggested interventions to complement implementation of the MSB. |
| Evidence Strength & Quality | The kind of supporting evidence or proof is needed about the effectiveness of maternal safety bundles to get staff on board, including involvement of co-workers and administrative leaders. |
| Administration or Senior Leadership | Support from leadership. |
| Joint Commission | Support for the MSB and associated measures by the Joint Commission |
| Other support points or stakeholders | Support for MSBs from other sources, except for joint commission, leadership and providers. |
| Providers (Physician, RN, PA, etc.) | Support by other providers. |
| **Outer Setting** |  |
| External policies and incentives | External factors that influenced the decision to implement the Equity MSB. |
| Financial incentives | Financial incentive to implementing Equity MSB. |
| Missing incentives | What incentives were missing from the first year of MSB implementation. |
| Performance measures | Local, state, or national performance measures as influences to deciding to implement the Equity MSB. |
| Policies, regulations, & guidelines | Policies (national/local) or other guidelines/regulations as influencing the decision to implement the Equity MSB. |
| Patient Needs and Resources | How well participants believe the implementation of the Equity MSB will meet the needs of the patients at their sites. |
| Pandemic's influence on implementation | How the COVID-19 pandemic influenced or impacted implementation of the Equity MSB. |
| **Implementation Process** |  |
| Executing | Carrying out or accomplishing the implementation according to plan. |
| Reflecting & Evaluating | Quantitative and qualitative feedback about the progress and quality of implementation accompanied with regular personal and team debriefing about progress and experience. |
| Gaps in implementation | Participants discussing what was left out of implementation - either in the bundle or in how it was implemented. |
| Difficulties in Implementation | Participants discussing difficulties they had with implementing components of the bundle |
| Siloes between RNs and MDs | Discussing siloes between physicians and nurses in practice, culture, and education. |
| Successes with implementation | Participants discussing what went well in implementing the Equity MSB. |
| Feedback | How will and what kind of feedback be collected by the institution on the impact of the Equity MSB. |
| Planning | The degree to which a scheme or method of behavior and tasks for implementing an innovation are developed in advance, and the quality of those schemes or methods. |
